# Case of Severe Wound of Abdomen

**Published:** 1851-08

**Authors:** P. G. Bertolet

**Affiliations:** Berks Co., Pa.


*Case of Severe Wound of Abdomen.—Recovery.* By P. G. Bertolet, M. D., Berks Co., Pa.

On the 20th of September, 1848, I was called to see H. G., æt. 12 years, who was gored by a bull at 8 o'clock, A. M., and reached the patient about an hour after the occurrence of the accident. His clothes were literally torn into shreds, and covered with blood and dirt, while he vomited almost incessantly frothy matter mixed with blood. On removing his clothes, the following injuries were presented:

On the left cheek was a lacerated wound, extending from the angle of the mouth towards the ear about one and a half inches. It bled very little. Other parts of the *head* and *face* were much bruised and swollen.

Upon the anterior and posterior surface of the *thorax* were several severe contusions.

About an inch anteriorly and a little below the angle of the twelfth rib, on the left side, was a lacerated wound of the *abdomen*, running almost directly forwards, one and a quarter inches in length. Through this opening in the abdominal parietes protruded a portion of lacerated *omentum*, the size of a man's hand, and ten inches of the *colon*, probably a portion of the sigmoid flexure of that intestine. (That it was this intestine, was obvious from the peculiar arrangement of its longitudinal fibres or bands.) The protruding portions of the omentum and gut were matted with the torn clothes, and covered with sand and grass.

The coats of the *colon* were, however, entire, with the exception that several portions of it were stripped of the peritoneal coverings, and in one spot much contused, causing thickening by infiltration and swelling. It was pretty firmly held by the lips of the wound, so as to cause partial strangulation.

It is probable that the horn entered the abdomen at the wound, passed obliquely downwards and backwards, and upon its withdrawal produced the condition of things described.

There were three other extensive lacerated wounds—one involving the *scrotum;* one the upper and posterior part of the *thigh;* and the other of the *nates.* These wounds were nearly in a direct line, and appear to have been made by a single gash with the horn obliquely across the nates and thigh of the boy, from right to left.

The wound of the scrotum was triangular in shape, one angle extending to the root of the penis, so that fears were entertained that the urethra might be injured. The left testicle was denuded of all its investments, leaving the organ bare and pendulous by its cord.

The two other wounds were deep lacerated flesh wounds; that on the *right buttock* severed the glutæi muscles throughout their depth, while the opposite one involved the heads of the posterior muscles of the thigh. The sciatic nerve was torn off at this place, and nervous threads were drawn out, some six inches in length; these lacerated and protruding fasciculi were cut away with the scissors.

Fortunately the lad, after receiving so many hard knocks and bruises, had no fractured bones. The lower extremities were severely bruised in several places, but nearly all the suffering was referred to the left leg.

The patient was insensible during the greater part of the investigation and dressing, muttering incoherent words, but at times he would rouse up as from fear. I have seen a number of surgical cases during an extensive rural practice of six years, but never did I discover life in such a mutilated human being. He was of a passive temperament and had a strong constitution.

After acquainting myself with the position of the patient, without delay the aid of Drs. Darrah and Filbert was sought, who arrived at 10 o'clock, during which time I busied myself in cleansing and washing the patient.

On *consultation*, it was concluded that by reducing the protruding viscera and dressing the wound upon general principles, and by observing the strictest precautions in the regimen, the best possible chance would be afforded to the patient.

The injuries of the abdomen required our first attention. The protruding viscera were carefully cleansed and reduced by taxis; but it was not until half an hour's patient and careful perseverance, that we succeeded in the reduction of the *colon*. It was then pushed back as far as possible with the point of the finger, in order to bring it to its natural position.

The protruding omentum was much lacerated, (so that it appeared like coarse fringe,) and these loose and pendulous shreds were removed with the scissors; and after bathing it with cold water, which restrained the oozing hæmorrhage, it was reduced, and the wound carefully closed with the interrupted suture and a few strips of adhesive plaster; over this was applied a compress, and the whole dressing was retained by a broad roller, which served at the same time to keep the parts at rest.

Immediately after the reduction of these organs, the vomiting ceased, and the patient became more rational. The pulse also became fuller and stronger, having been previously very small and weak.

Next we introduced a catheter into the bladder, in order to ascertain the continuity of the *urethra*, for which fears were entertained; it however passed readily. The wound of the *scrotum* was closed by bringing together the loose coverings over the testicle to the root of the penis, and the edges retained by the glover's suture.

The wounds of the nates and thigh were also closed with sutures, in order to obtain all possible immediate union; and to give permanency to the sutures, adhesive strips were applied over them. A layer of charpie, smeared with simple ointment, was applied over this as well as that of the scrotum; and all these dressings were retained with several rollers.

The laceration of the lip was closed with the twisted suture.

It was now 12 o'clock, M., and the wounds of our patient were all dressed; he was now placed in a clean bed, loosely covered with a sheet or two, after which he expressed himself more comfortable.

The strictest precaution was given to the nurse to have the patient kept absolutely quiet, both mentally and corporeally. Diet, about a teacupful of rice water per day, and an opiate, if he should at any time become very restless.

*Sept. 21st.*—Eight o'clock, A. M. Found patient conscious and cheerful; complains of little pain, except in his left leg; he has slight tenderness on the right side of the abdomen, but none around the wound on pressure; some distention, or at least tendency to meteorism; vomited none; slept several hours during the night. Pulse 102 per minute. Thirst moderate. Tongue a little furred along its centre. Urinated freely—urine of limpid color. Had no alvine evacuation. Wounds gave patient little suffering and discharged very little.

It was deemed prudent to defer moving the bowels as long as possible; but directions were given, if the flatulency and colicy pains should become more troublesome, they should give two drachms of castor oil with twenty drops of laudanum, to be repeated every three hours alternately with enemata consisting of milk and water, half a pint, with castor oil, to be continued until relief was afforded or the bowels opened.

The dressings were left untouched and the rest of the treatment continued.

Seven o'clock, P. M. Found that the patient had vomited all medicines given after the use of the first enema, and the bowels were not opened. The abdomen greatly distended and sensitive to pressure. Patient is weak, delirious and restless; pulse small and 140 per minute; took no nourishment during the day; vomited bilious matter all the afternoon.

Continued the enemata with some laudanum added, and after several injections the patient became composed.

22*d.*—Nine o'clock, A. M. Patient rational; distention of abdomen much diminished; has little pain, except in his right side (of the abdomen;) pulse soft and 88 per minute; partook of rice water this morning; vomited none since 4 o'clock; glysters brought away some feculent matter; urinated none for fifteen hours; drew away with the catheter about a pint of urine, which greatly relieved the pain in his abdomen.

Seven o'clock, P. M. Continues the same as in the morning.

23*d.*—Half past ten o'clock, A. M. General appearance of patient good, but has more thirst; tongue dry and red along its centre; surface somewhat hotter than natural, but skin feels moist; slept some during the night; complains of little pain, but nausea; voided some alvine matter with injections; urinated freely; wounds begin to suppurate. Treatment, with the omission of the glysters, was continued.

24*th.*—Nine o'clock, A. M. Found patient cheerful; pulse 75 per minute; respiration free; skin moist; less thirst; tongue moister and softer than yesterday; says he has no pain, even not in the abdomen under moderate pressure; tympanitis disappeared, and had the first natural evacuation this morning. Passed a comfortable night, and was disturbed only once, while dreaming about the *bull;* calls for more nourishment. The wounds were suppurating freely; found it necessary to change (for the first time) the dressings. The greater portion of the wound of the abdomen had already united, leaving open only its posterior extremity, which discharged healthy pus, with some sanious fluid. The wounds in general looked well, and the simple dressing was reapplied. The wound of the cheek being united, the needles were withdrawn. Treatment continued.

*25th.*—General condition of patient as yesterday. Tongue begins to clean off.

*26th.*—Patient says he feels well, and asks for more liberal diet.

*27th.*—Gave a dose of castor oil, which brought away a copious stool containing fine dead lumbricoides; felt well on the evacuation. Wounds discharge freely; craves more food. Directed the addition of one cracker per day to his bill of fare, and substitute a pill of Ex. Hyoscyam. at bed time in place of the opiate.

*28th.*—Patient doing well; wounds begin to granulate finely, except that of the scrotum, which is somewhat indurated; emollient poultices to be applied to this.

The bowels were deranged from time to time, but were speedily brought to act by means of a little oil or a glyster or two.

*30th.*—Doing well; ordered some chicken broth.

*Oct. 2d.*—Found patient doing so well, that I deemed it superfluous to continue regular visits, but left strict orders that he should not assume the erect posture too soon, &c.

*Nov. 11th.*—I was again requested to see this boy, and upon examination found a prominence of the abdominal walls, several inches anterior to the place of the wound. The tumor measured about two inches in diameter at its base, while its surface was elevated about three-fourths of an inch above the surrounding parts. This condition of things was undoubtedly brought about by the patient's own imprudence, by assuming the erect posture sooner than was compatible with either his physician's directions or his own safety. The walls of the abdomen were not yet sufficiently strong to bear against the loaded viscera, and yielded under the pressure.

A truss with a large pad and easy spring, made expressly for him, relieved the case, and his protrusion has long since disappeared, and he has dispensed with his truss.

During the treatment the patient complained of *much* pain in his left leg, though some of the most important nervous trunks of this limb were severed. He slowly regained the use of the limb, the motion of it being at first very imperfect, but in the course of several months regained its perfect use. The lad is now in excellent health.

The patient has recovered so perfectly, that a suit entered by his friends against the owner of the bull, was decided in favor of the defendant, the legal body finding nothing for which to allow plaintiff any damages.

